# The Effect of Levagen+ (Palmitoylethanolamide) Supplementation on Symptoms of Allergic Rhinitis—A Double-Blind Placebo-Controlled Trial

**DOI:** 10.3390/nu15234940

**Published:** 2023-11-28

**Authors:** David Briskey, Phillippa Ebelt, Amanda Rao

**Affiliations:** 1 RDC Clinical, Brisbane, QLD 4006, Australia; d.briskey@uq.edu.au (D.B.);; 2 School of Human Movement and Nutrition Sciences, University of Queensland, Brisbane, QLD 4072, Australia

**Keywords:** Palmitoylethanolamide, Levagen+, allergic rhinitis, histamine, inflammation, reflective total nasal symptom score

## Abstract

Background: Allergic rhinitis (AR) is an inflammatory, symptomatic disorder stimulated by antigen-specific immunoglobulin E inflammation in response to allergens. Current treatments include the use of corticosteroids and antihistamines to reduce inflammation by preventing histamine release. Palmitoylethanolamide (PEA) is reported to be an alternative treatment, shown to downregulate mast cell activation and increase the synthesis of endocannabinoid 2-Arachidonoylglycerol to reduce histamine and the symptoms of AR. Method: A double-blind, randomised, placebo-controlled clinical trial in which 108 participants presenting with seasonal AR were supplemented with either 350 mg of PEA (Levagen+) or a placebo daily for two weeks. Symptom scores were recorded using the reflective total nasal symptom score (rTNSS) twice a day (morning and evening) for the two weeks, and blood was taken at baseline and week 2. Results: 101 participants completed the study with no baseline group differences. No significant difference was seen between groups for allergy symptoms scores (rTNSS) throughout the 14 days of treatment. A sub-group analysis of participants scoring over four (mild-to-moderate) on the total rTNSS at baseline showed that Levagen+ significantly reduced scores compared to the placebo group. Only 36 participants had full sets of blood taken due to COVID-19. The pathology results showed a significant difference in change from baseline between groups. The Levagen+ group had a significant decrease from baseline in histamine, IL-4, IL-8, IL-10, and TNF-α. The placebo group only had a reduction in IL-4. Conclusion: The results of this study show that Levagen+ can alleviate AR symptoms, resulting in a reduction in histamine and inflammatory markers.

## 1. Introduction

Allergic rhinitis (AR) is an inflammatory, symptomatic disorder triggered by antigen-specific immunoglobulin E (IgE) inflammation in response to allergen exposure in the nasal mucosa [1]. As the most prevalent form of allergic respiratory conditions, AR affects 15% of the Australian population and 40% worldwide, with the highest incidence among adults aged 25 to 44 years old [2,3]. This age-dependent onset is believed to be driven by gradual cellular aging and immune dysfunction or modifications that occur during this period [4]. Clinically, AR is characterized by symptoms such as sneezing, nasal congestion, itching, and a runny nose [5]. AR symptoms are also associated with pathogenic responses linked to conditions like asthma, sinusitis, nasal polyposis, and lower respiratory tract infections [6].

Allergic rhinitis exhibits a complex pathophysiology that encompasses various molecular mechanisms and the involvement of inflammatory and immune cells. Both acute and chronic reactions in AR are orchestrated by the binding of allergens, such as pollen or mites, to IgE receptors situated on mast cells and basophils [7]. This interaction, in turn, triggers an inflammatory cascade, leading to the release of pro-inflammatory mediators, including histamine, leukotrienes, and tryptase, into the nasal mucosa [8].

Among the pro-inflammatory mediators, histamine assumes a central role in AR, primarily responsible for activating sensory nerve endings in nasal nociceptors and prompting the release of acetylcholine from nasal parasympathetic nerves. This acetylcholine release, in turn, leads to increased mucus secretion, contributing to nasal congestion and a runny nose [8]. Histamine also facilitates the activation of H1 receptors located on sensory nerves, giving rise to symptoms such as sneezing and itching [9]. Reactions that develop a few hours after allergen exposure are referred to as the late-phase response and involve the release of pro-inflammatory cytokines and other immune cells from resident nasal mast cells and basophils. The influx of these various cell populations further fuels the allergic response and exacerbates symptom manifestation [10].

Following exposure to an allergen, histamine is one of the main contributors to initiating an immune response [11]. Increased plasma histamine has been shown during both severe allergic reactions and in experimental allergy models [12]. An elevation in histamine correlates with the level of mast cell activation and release of proinflammatory mediators [13,14]. This complex interplay of immune cells, mediators, chemokines, cytokines, and the external environment results in the development of allergic rhinitis and its clinical manifestations [15]. Currently, standard pharmacological treatments rely on the use of intranasal corticosteroids and oral antihistamines, both of which work to reduce inflammation by preventing histamine release [16].

The intricate nature of AR pathophysiology has presented challenges in its treatment, leading to the development of various therapeutic options, including the emergence of alternative natural treatments aimed at addressing and preventing AR symptoms. As hypersensitivities become more prevalent, conventional treatments like antihistamines primarily focus on inhibiting the binding of histamine released from mast cells to histamine receptors to help prevent the inflammatory response triggered by IgE mast cell activation [17]. Palmitoylethanolamide (PEA), a naturally occurring lipid molecule, is gaining recognition for its potential therapeutic benefits in various inflammatory and neuropathic conditions, including AR.

PEA has been reported to downregulate mast cell activation and inhibit the release of mast cell mediators, including histamine [18]. This naturally occurring endogenous compound has been shown to limit mast cell activation by reducing the release of β-hexosaminidase and serotonin triggered by IgE receptor crosslinking [19]. PEA has also demonstrated the ability to increase β-enzyme activity, which, in turn, enhances the synthesis of endocannabinoid 2-arachidonoylglycerol, suggesting that an increased endocannabinoid tone may modulate mast cell degranulation [19,20]. Together, these effects seen in cells suggest that PEA supplementation in humans may have the potential to prevent the release of histamine from mast cells when triggered by IgE in response to an allergen.

However, both endogenous levels of PEA and exogenous PEA administration have previously been reported to be insufficient to mitigate a significant inflammatory response due to poor absorption, resulting in a low plasma concentration [21,22]. When PEA is combined with dispersion technology, its absorption can be significantly increased, leading to higher plasma concentration levels that may enable a therapeutic effect [23]. Despite some theoretical support for the use of PEA in AR, further research is needed to establish its effectiveness and safety for this specific condition.

To the best of our knowledge, there have been no clinical studies to date that specifically investigate the impact of PEA on allergy symptoms, such as AR. One study by Antonucci (2015) supplemented a child with autism and allergy symptoms, including rhinitis, with 600 mg of PEA daily for one month and was able to show improvements in allergy symptoms, although there were no differences in IgE levels [24]. An animal study conducted by Roviezzo and colleagues on mice found that PEA, dosed at 10 mg/kg, prevented bronchial hyperactivity and allergen-induced cell recruitment in asthma [25]. Whilst this study is not specific to allergy rhinitis, mast cells are still recruited in this process, and PEA was able prevent the release of these pro-inflammatory cells. 

Therefore, building on prior research and recognizing the potential of PEA as a therapeutic choice for individuals with AR, the aim of the present study was to assess the impact of a PEA supplement enriched with dispersion technology to aid absorption (Levagen+) in alleviating allergy symptoms in comparison to a placebo group during a 14-day treatment period. Our hypothesis is that the administration of Levagen+ will result in a reduction in the severity of allergy symptoms when compared to a placebo over the course of a 2-week intervention period.

## 2. Methods

A double-blind, randomised, placebo-controlled clinical trial with a 2-week supplement period was carried out. Sample size calculations indicated 50 participants were required per group for the power to detect a change of 2.0 in the reflective total nasal symptom score (rTNSS) between groups (2.8 ± 3.0 vs. 4.8 ± 3.0) at the completion of the trial. The calculated effect size was 0.667; the alpha error probability was 0.05; and the power was 0.95. This study was conducted with ethics approval from the Bellberry human research ethics committee (approval number 2019-05-405) and registered with the Australian New Zealand Clinical Trials Registry (ACTRN12619001368123).

This study involved two groups: an active group receiving Levagen+ and a placebo group. Levagen+ was provided by the manufacturer (Gencor Pacific, Hong Kong) and encapsulated under good manufacturing practice conditions. Each Levagen+ capsule contained 175 mg of PEA and 30 mg of excipient, and each placebo capsule contained 175 mg of maltodextrin. Both groups took two capsules daily in the morning, resulting in a total daily dose of 350 mg of PEA or maltodextrin. All trial products were enclosed in opaque capsules that were indistinguishable in appearance and administered in a consistent manner.

Potential participants underwent a telehealth screening process before enrolment, which involved assessing them against specific inclusion and exclusion criteria. To be eligible for inclusion, participants needed to be 18 years of age or older, in generally good health, capable of providing informed consent, experiencing seasonal allergic rhinitis, and agreeable to maintaining their existing diet and exercise routine and refraining from using any alternative allergy relief supplements or products during the study. Female participants were required to be on a prescribed form of birth control, practicing abstinence, or post-menopausal. Additionally, all participants were required to achieve a minimum score of 3 or higher on at least 4 of the 7 baseline days for the 24 h score on the rTNSS, following the granting of consent. The rTNSS consists of 5 questions rating participants symptoms for (1) nasal congestion, (2) runny nose, (3) nasal itching, (4) sneezing, and (5) sleep difficulty over the last 12 h. Each symptom domain is scored using a four-point scale (Table 1). The total rTNSS score is calculated by adding the score for each of the symptoms at each time point to obtain a total out of 15. A total score of 0–3 was classed as mild, 4–9 mild-to-moderate and 10–15 moderate-to-sever.

Exclusion criteria encompassed individuals with allergies to any of the components in the active or placebo formulas, a history of chronic alcohol consumption (more than 14 alcoholic drinks per week), active smoking, substance abuse, pregnancy, or lactation. Furthermore, those with unstable or severe medical conditions, including multiple sclerosis, renal, liver, gastrointestinal, cardiovascular, diabetes, endocrine disorders, or mood disorders, such as depression and bipolar disorder, malignancies, or recent treatment for malignancies within the past two years, were excluded. Also, participants who were currently receiving or prescribed anticoagulation therapy like warfarin, heparin, dalteparin, enoxaparin, or other similar medications were ineligible for participation in the study.

Eligible participants attended the study clinic for an information session before providing their written consent for inclusion in the trial. Following consent, participants underwent a health assessment that included lifestyle, current medications, and medical history. Consenting participants first recorded their symptoms twice a day for 7 days to establish rTNSS baseline scores prior to supplementation. Participants that scored 3 or more on at least 4 of the 7 baseline days for the 24 h score on the rTNSS were invited to return to the clinic and randomly assigned, via random allocation software (www.sealedenvelope.com, accessed on 19 September 2019), to either the active or placebo group. All trial participants, investigators conducting the trial, the biochemist analysing the samples, and the statistician were blinded to allocation. During the randomisation clinic visit, participants underwent baseline measurements, including the Rhinoconjuctivitis Quality of Life Questionnaire (RQLQ) and a 20 mL blood sample to measure pathology markers. Pathology markers were assessed for change in allergy/inflammatory response markers (histamine, cytokines, hs-CRP) and safety (full blood count (FBC) and enzyme and liver function testing (E/LFT)).

After receiving the product, participants were instructed to take it daily for a period of two weeks. During this time, they were also asked to complete the allergy symptom questionnaire (rTNSS) online every morning and evening. At the end of this 2-week treatment period, participants had a follow-up evaluation, which involved repeating the baseline measurements (rTNSS, RQLQ, and bloods).

The primary outcome of the study was assessed by measuring the change in allergy symptoms, specifically the total rTNSS score after 12 h, which included components related to nasal congestion, sneezing, an itchy nose, and a runny nose. Secondary measurements included individual rTNSS scores, the overall score on the RQLQ (Rhinoconjunctivitis Quality of Life Questionnaire), changes in biochemical markers from baseline (cytokines, hs-CRP, FBC, histamine, and E/LFT), and the onset of action, indicating when participants first noticed an improvement in their symptoms.

The analysis was conducted using either GraphPad Prism 8.0 or SPSS 25. All results were first tested for normality before any other test was conducted. Based on the distribution of the data, the appropriate statistical tests were used. In a parametric scenario, paired t-tests were used to analyse within-group differences from baseline to the endpoint. Repeated-measures ANOVA/ANCOVA were used to analyse within-group variables from all time points, with and without covariates. A general linear mixed modelling was also performed on participant data to compare group dynamics. Results were considered statistically significant if *p* < 0.05.

## 3. Results

Overall, a total of 108 participants presenting with seasonal allergic rhinitis were randomised from the 23 September 2019 to the 11 February 2022. Of the randomised participants, 101 (92.5%) otherwise healthy male and female participants aged between 21 and 77 years completed the study (Table 2). Four participants were lost to follow-up, one participant was dropped from the study, and three participants within the placebo group reported adverse events (1 × bloating, 1 × diarrhoea, and 1 × mild chest pain). There was no significant difference between the groups in age, treatment groups, or baseline measurements (Table 2).

**Figure 1:**
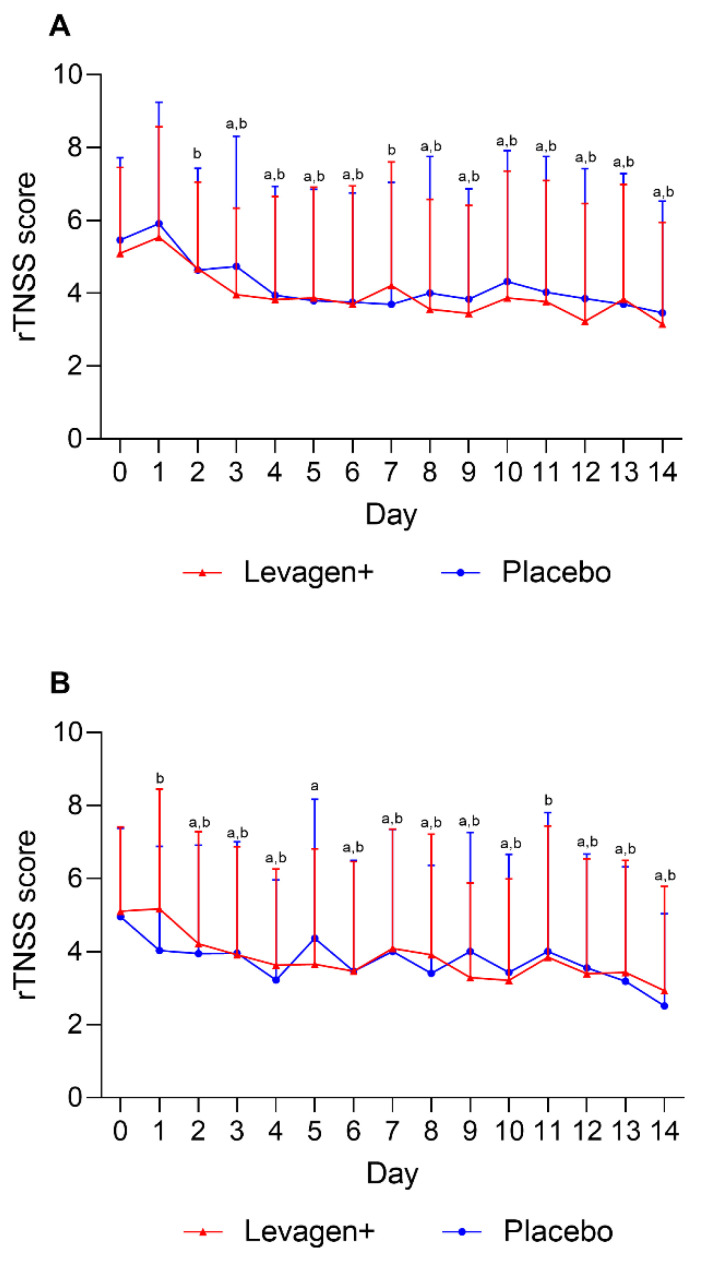
Daily reflective total nasal symptoms (rTNSS) scoring for (**A**) AM scores and (**B**) PM scores. ^a^ = significant difference (*p* < 0.05) to baseline scores for Levagen+ group; ^b^ = significant difference (*p* < 0.05) to baseline scores for placebo group.

No significant difference was seen between the Levagen+ and placebo groups for the allergy symptoms scores (rTNSS) at baseline or throughout the 14 days of treatment (Figure 1). Both groups had decreased rTNSS symptom scores from day 3 for morning scores and day 2 for evening scores (Figure 1). 

**Figure 2:**
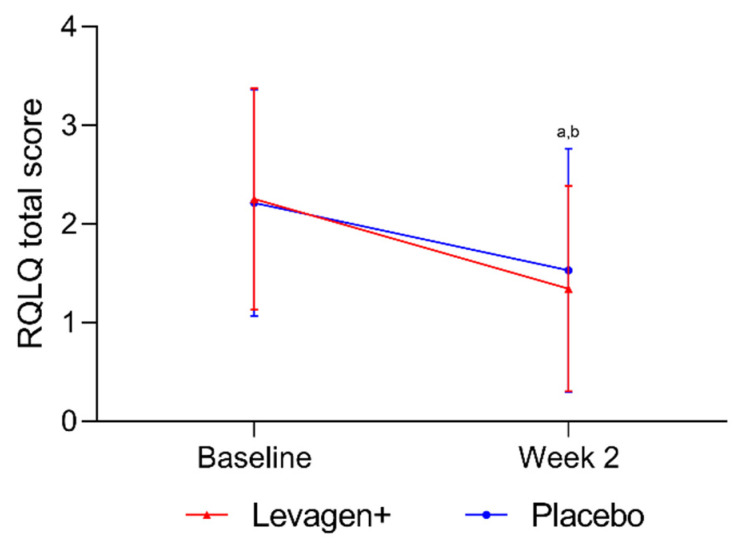
Total RQLQ scores for all participants. ^a^ = significant difference (*p* < 0.05) to baseline scores for Levagen+ group; ^b^ = significant difference (*p* < 0.05) to baseline scores for placebo group.

No significant difference was seen between the groups for the total RQLQ score from baseline to week 2 (Figure 2). Both groups had a significant reduction from baseline in total RQLQ scores. Both groups also reported a significant reduction in all domains of the RQLQ over the 2 weeks, but there were no between-group differences.

**Figure 3:**
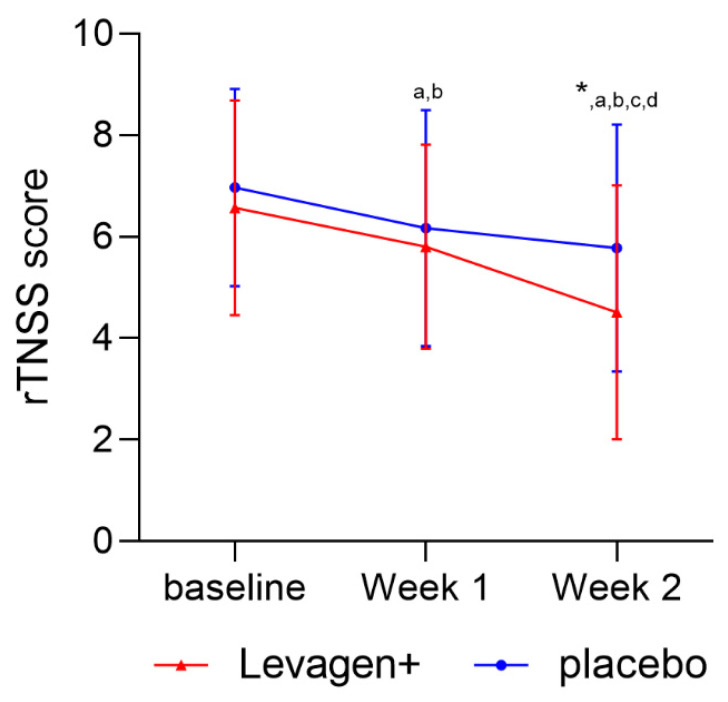
Weekly TNSS score (day + night) for sub-group scoring over 4 (mild-to-moderate nasal symptoms) on the rTNSS at baseline. * Significant change from baseline; ^a^ = significant difference (*p* < 0.05) to baseline scores for Levagen+ group; ^b^ = significant difference (*p* < 0.05) to baseline scores for placebo group; ^c^ = significant difference (*p* < 0.05) to week 1 scores for Levagen+ group; ^d^ = significant difference (*p* < 0.05) to week 1 scores for placebo group.

The sub-group analysis conducted on the participants scoring over four on the total rTNSS weekly score (n = 79) showed a significant reduction in both groups over the two weeks, with a significant difference between the groups at week 2 of treatment (Figure 3).

Due to the COVID-19 pandemic, not all the participants were able to attend the clinic for pathology testing. The results presented are for the participants who were able to complete both baseline and final blood draws (n = 36). A significant difference was seen between groups for the change in plasma histamine concentration (Table 3). IL-4 significantly decreased from baseline for both groups. Plasma histamine, IL-10, IL-8, and TNF-α significantly decreased compared to baseline in the Levagen+ group only (Table 3).

## 4. Discussion

Palmitoylethanolamide (PEA) is widely presented in the literature for its analgesic and anti-inflammatory activity for the management of multiple clinical conditions [26]. In the context of allergic diseases, PEA functions as a mast cell stabiliser and inhibits histamine release [19]. Under continued cellular stress, PEA is upregulated and recruited to the inflammatory site in response [27]. This profound protective role suggests that PEA at higher concentrations can be advantageous for treating inflammatory and allergic conditions.

This study aimed to evaluate the effect of Levagen+ for alleviating allergy symptoms compared to a placebo over a 14-day supplementation period, as measured by the rTNSS. However, the results indicate that there was no significant difference between the Levagen+ and placebo groups in terms of rTNSS scores at baseline or throughout the treatment period. Both groups exhibited a decrease in symptom scores over the 2 weeks, with improvements beginning as early as day 3 for morning scores and day 2 for evening scores. The lack of difference between groups warrants a deeper examination of the study’s findings.

One effect to consider is the potentially strong influence of the placebo effect. The fact that both groups showed improvements in symptom scores suggests that psychological factors, such as participants expectations, may have played a substantial role in symptom relief. This highlights the importance of using a robust control group to differentiate the effects of a treatment from those of a placebo.

Another consideration is the natural course of allergic symptoms. Allergies typically fluctuate in symptom severity due to their influence by environmental allergen exposure, with both groups showing almost immediate symptom relief, which could be attributed to allergen exposure levels or a strong placebo effect. The results suggest the need for longer-term studies to assess the potential efficacy of Levagen+, or any potential treatment, for allergies to help account for the natural variation in symptoms and any possible placebo effect.

With the overall results indicating that both groups had reduced symptoms, participants were sub-grouped to only include those with symptoms classified as a minimum of mild (a score of four or greater) on the baseline rTNSS scores. The sub-group analysis of those with greater symptom scores at baseline showed that Levagen+ led to a significant reduction in symptom scores compared to the placebo at week 2 of treatment (Figure 3). The results of the sub-group analysis support a clinical case study showing that 4 weeks of supplementation with PEA reduced allergic reactions (nasal itching and nasal oedema) [24]. The results of Antonucci’s study also shows that a longer duration may be required to see the best results from a study on allergy symptoms [24].

The sub-group analysis suggests that Levagen+ may be more beneficial for individuals with a greater severity of AR symptoms. Currently, mild, and moderate/severe allergic rhinitis have separate treatment plans using either antihistamine (for mild) or corticosteroids (for moderate/severe) [28]. Corticosteroids have been established to treat moderate or severe symptoms by stimulating endocannabinoid synthesis and signalling [29]. Similarly, PEA (Levagen+) modulates endocannabinoid signalling and contributes to the activation of cannabinoid receptors [19,20]. This may help explain why those with more severe symptom scores appear to respond better than those with mild symptoms. The aetiology of symptoms in those classified as less than mild may be unsuitable for PEA mechanisms to act on. 

To better explore the potential antihistamine and corticosteroid functions of PEA, blood was collected for an analysis of histamine and inflammation. However, one of the main limitations of this study is that it was conducted during the COVID-19 pandemic. This resulted in participants being unable or unwilling to attend the clinic to have blood samples collected. Of the 101 participants that completed the study, only 36 had blood collected at both the baseline and final points. The low number of participants likely reduced our ability to detect some significant changes in the blood. Despite this, there was a significant difference in the change in plasma histamine detected between the groups. The Levagen+ group showed a reduction in plasma histamine, while the placebo group had an increase. The significant difference in change in plasma histamine supports the notion that Levagen+ supplementation downregulates mast cell activation and thereby inhibits histamine release. The increase in histamine in the placebo group, despite the reduction in reported symptom severity, may indicate a continuation of AR. The findings of this study support the results of Petrosino and colleagues, showing PEA’s mechanistic control to downregulate mast cell activity and histamine release [19]. 

Along with the histamine reduction, we also showed that plasma pro-inflammatory IL-4, IL-8, and TNF-α and anti-inflammatory marker IL-10 were significantly reduced in the Levagen+ group, but only plasma IL-4 was significantly reduced in the placebo group. The reduction in the pro-inflammatory markers suggests that less inflammation is present, potentially due to the prevention of histamine release from mast cells. The reduction in anti-inflammatory IL-10 suggests that there is no need for an anti-inflammatory cytokine due to a lack of inflammatory signalling. Together, these cytokines play a central role in the pathogenesis of allergic rhinitis by promoting an allergic immune response, inflammation, and the release of allergic mediators. When activated, these cytokines can initiate the production of IgE antibodies and the recruitment of other immune cells, like eosinophils, involved in allergic reactions. This reaction can increase inflammation at the site where the immune reaction has occurred to help combat the noxious stimulus, and it generally results in AR symptoms (i.e., sneezing, nasal congestion, itching, or a runny nose). TNF-α, in turn can amplify the inflammatory response by promoting the activation of other immune cells and the release of additional proinflammatory cytokines. This can further exacerbate the symptoms of AR. Therefore, a reduction in rTNSS symptom scores, along with a reduction in histamine and cytokine production, suggests that Levagen+ may alleviate AR and prevent the release of histamine and subsequent inflammatory responses.

There were potentially several limitations to this study that were not foreseen when designing the study. First is the study duration. A 2-week supplementation period was designed, as we felt if the supplement did not work within 2 weeks, it was not worth using. However, we did not factor in, nor foresee, the seemingly strong placebo effect. Possibly a 4- or even 6-week intervention period is required to account for any potential placebo effect or fluctuation in allergy symptoms. 

A further limitation of this study, as previously mentioned, is that it was conducted during the COVID-19 pandemic. This circumstance resulted in reduced physical activity and potentially lower allergen exposure due to decreased external mobility. Consequently, these factors may have mitigated the severity of participants’ allergic responses, thereby potentially impacting the trial outcomes. Additionally, the increased amount of time individuals spent at home during the pandemic likely facilitated better symptom recovery, possibly contributing to the observed placebo effect.

Moreover, owing to the COVID-19 restrictions, we encountered challenges in collecting the desired amount of blood samples. Expanding the blood sample collection and exploring a wider range of inflammatory pathways related to allergic rhinitis (AR) could offer valuable insights into the aetiology of how Levagen+ alleviates AR symptoms.

The population selected for this study may have certain limitations. Although participants were screened for a minimum score on the rTNSS, it is possible that the threshold we set was not sufficiently high to consistently confirm the presence of an allergic rhinitis (AR) episode. In future studies, it could be advantageous to include participants from various severity classifications, such as mild, moderate, and severe, to assess the efficacy of Levagen + more comprehensively across different AR severities. Additionally, incorporating a more clinical diagnosis approach in future studies would help ensure that the reported symptoms align accurately with AR, enhancing the study’s reliability.

## 5. Conclusions

Despite the potential limitations of the study, the results show that Levagen+ can alleviate AR symptoms, resulting in a reduction in histamine and inflammatory markers. However, due to the strong placebo effect seen, more work is required to be able to definitively establish the full effect of Levagen+. Future clinical studies would benefit from including a longer study period.

## Figures and Tables

**Table 1:** Individual symptom rTNSS score scale.

Score	Severity	Symptoms
0	None	No symptoms evident
1	Mild	Symptom present but easily tolerated
2	Moderate	Definite awareness of symptom; bothersome but tolerable
3	Severe	Symptom hard to tolerate; interferes with daily activity

**Table 2:** Baseline characteristics for participants who completed the trial.

	Levagen+	Placebo
Participants (n)	52	49
Females (n)	34	38
Males (n)	18	11
Average age (years)	45.6 ± 15.6	45.3 ± 12.8

Data shown as mean ± SD.

**Table 3:** Change in biochemistry markers from baseline.

	Levagen+			Placebo		
	Baseline	Week 2	Change	Baseline	Week 2	Change
P histamine (ng/mL)	0.23 ± 0.11	0.17 ± 0.09 ^a^	−0.05 ± 0.11 *	0.16 ± 0.11	0.20 ± 0.15	0.04 ± 0.13
WB histamine (ng/mL)	18.14 ± 12.44	16.42 ± 10.65	−1.92 ± 6.69	15.94 ± 15.48	19.62 ± 15.62	3.34 ± 9.97
IL-10 (pg/mL)	4.26 ± 3.37	3.13 ± 2.55 ^a^	−1.13 ± 1.56	5.74 ± 6.61	4.55 ± 5.97	−1.19 ± 2.38
IL-4 (pg/mL)	124.5 ± 189.2	107.7 ± 174.6 ^a^	−16.8 ± 26.9	105.3 ± 185.9	89.9 ± 167.3 ^a^	−16.7 ± 21.8
IL-6 (pg/mL)	8.07 ± 16.19	7.07 ± 14.07	−1.00 ± 2.87	6.59 ± 12.03	5.99 ± 11.19	−0.60 ± 1.00
IL-8 (pg/mL)	17.57 ± 24.48	14.99 ± 22.83 ^a^	−2.58 ± 2.44	8.92 ± 9.26	6.86 ±6.39	−1.91 ± 5.19
TNF-α (pg/mL)	5.85 ± 2.73	4.70 ± 1.84 ^a^	−1.16 ± 1.68	8.08 ± 5.65	6.44 ± 4.94	−1.70 ± 4.16

Values are mean ± SD; * significant difference (*p* < 0.05) between groups. ^a^ Significant difference compared to baseline within the same group.

## Data Availability

Data are available from the corresponding author on reasonable request.
